# Similar or Different Neuropsychological Profiles? Only Set Shifting Differentiates Women With Bipolar vs. Borderline Personality Disorder

**DOI:** 10.3389/fpsyt.2021.690808

**Published:** 2021-07-28

**Authors:** Ioannis Michopoulos, Kalliopi Tournikioti, Antonios Paraschakis, Anna Karavia, Rossetos Gournellis, Nikolaos Smyrnis, Panagiotis Ferentinos

**Affiliations:** ^1^2nd Department of Psychiatry, Medical School, National and Kapodistrian University of Athens, “Attikon” Hospital, Athens, Greece; ^2^Psychiatric Hospital of Attica “Dafni”, Athens, Greece

**Keywords:** bipolar, borderline, CANTAB, executive function, memory, set shifting

## Abstract

There is ongoing debate about the similarities and differences between bipolar disorder (BD) and borderline personality disorder (BPD). Very few studies have concurrently assessed their neuropsychological profile and only on a narrow array of neuropsychological tests. We aimed to investigate the differences of these two patient groups on visual memory, executive function, and response inhibition. Twenty-nine BD patients, 27 BPD patients and 22 controls (all female) were directly compared on paired associates learning (PAL), set shifting (ID/ED), problem solving (SOC), and response inhibition (SSRT) using Cambridge Neuropsychological Test Automated Battery (CANTAB). Rank-normalized outcomes were contrasted in one-way ANOVA tests. Discriminant analysis was finally performed to predict BD or BPD patient status. BD patients performed significantly worse than controls on all tasks. BPD patients performed significantly worse than HC on all tests except SST. Significant differences between the two patient groups were recorded only on ID/ED, where BPD patients performed worse (*p* = 0.044). A forward stepwise discriminant analysis model based on ID/ED and SOC predicted correctly patients' group at 67.9% of cases. In conclusion, BD and BPD female patients appear to be more similar than different as regards their neuropsychological functions. This study is the first to show that BPD patients display more deficits than BD patients when directly compared on the set shifting executive function test, a marker of cognitive flexibility. Discerning BD from BPD patients through neuropsychological performance is promising but would improve by using additional subtler tests and psychometric evaluation.

## Introduction

There is a debate if BPD is a specific syndrome or should be a part of bipolar disorder spectrum disorders (1). Bipolar disorder (BD) and Borderline personality disorder (BPD) share predisposing factors, clinical features, and similar response to medication with mood stabilizers (2–4). Childhood trauma and family history of bipolarity are two common factors found in both BD and BPD (3). Regarding clinical features, they both present with mood dysregulation, impulsivity, suicidality and psychotic, or dissociative symptoms (4). Even though most of the clinical features have different duration or severity in BD and BPD, two of them, mood fluctuation, and impulsivity, make the distinction among the two entities especially challenging (3–5). A plethora of studies have compared either BD or BPD patients with healthy controls (HC) but only few have directly compared the neuropsychological profile of the two patient groups (2, 6), even though this could lead to valuable insights on their pathophysiology and management.

The relevant literature shows that both BD and BPD patients present neuropsychological deficits in comparison to HC. A common proposed etiological pathway implicates childhood trauma, a condition often shared by both entities (2). BD patients compared to controls show impaired neuropsychological performance. They have memory deficits, present even in euthymic patients, more prominent in verbal than visuo-spatial memory. They show attentional abnormalities in sustained attention and inhibitory control, persisting in remission. Executive function is also affected: planning, abstract concept formation and set shifting are impaired, though it could improve in euthymic patients (7). Meta-analytic studies have proposed that deficits involving attention, memory and executive function could be trait-related in BD (8). Meta-analytic studies have shown that BPD patients also perform poorer in neuropsychological testing, compared to controls. BPD patients show abnormalities in attention and speed processing, learning and memory (verbal and visuo-spatial), cognitive flexibility and planning (9). A most recent meta-analysis has proposed that these deficits could be dependent on co-morbidity, especially major depression, eating disorders, substance abuse, and other co-existing personality disorder (10). Impairments in memory and executive functions seem the most replicated findings across all mood states of BD (11), whilst patients with BPD appear to perform more poorly than HC in neurocognitive domains such as memory, attention, language, and executive functions (12–15).

As previously mentioned, what the literature mostly misses are direct comparisons among BPD and BD patients regarding their neuropsychological functions (16). Furthermore, it is not clear whether gender plays a role in the presented deficits, with differences being more or less accentuated among same-sex patients of the two disorders.

Our aim was to contribute to this apparent lack of data. We aimed to compare two groups of female patients, one with BD and the other with BPD, as well as to a group of HC. We focused on several neuropsychological domains: paired associates learning, as a marker of visuo-spatial memory, problem solving as a marker of planning, set shifting as a marker of cognitive flexibility and response inhibition as a marker of impulsivity. Our hypothesis was that BPD and BD patients would have differences in their neuropsychological profile.

## Methods

### Participants

Fifty-six female patients were consecutively recruited from the psychiatric inpatient unit of the 2nd Department of Psychiatry at “Attikon” General Hospital during a 2-year period. Twenty-nine patients had a lifetime BD type I diagnosis based on SCID-5-CV (17) and twenty-seven a BPD diagnosis based on SCID-5-PD (18). All patients were assessed close to discharge after having responded to treatment while at least partially remitted. Patients were excluded if they had a serious neurological and/or medical condition or a history of substance/alcohol misuse in the past 6 months. Finally, BPD patients diagnosed with major depressive episode in the past 6 months or lifetime BD (type I, II, other or unspecified) or schizophrenia/ schizoaffective disorder based on SCID-5-CV were also not comprised. No other comorbid psychiatric diagnoses were exclusion criteria for either group. Out of a total of 63 patients initially screened, seven patients were finally excluded; two BD patients and three BPD patients with substance/alcohol misuse in the past 6 months as well as two patients meeting criteria for both BD and BPD. No patient in either group met criteria for any other personality disorder. Any other comorbid psychiatric diagnoses apart from those used for exclusion were not systematically recorded for either group. Twenty-two (19) female HC with no psychiatric history and no current serious medical condition served as a comparison group.

Every participant signed an informed consent form in order to participate in the study. The Ethics Committee of “Attikon” General Hospital approved the study (ΨYX, EBΔ654/01-10-2018) which was completed in accordance with the Helsinki Declaration.

### Procedure

Clinical assessment and symptom evaluation took place on the same day of cognitive testing using the 17-item Hamilton Depression Rating Scale (HAMD-17) and the Young Mania Rating Scale (YMRS). We administered a series of tests from the Cambridge Neuropsychological Test Automated Battery (CANTAB), which is a computer-administered set of tasks developed to assess specific components of cognitive function (20, 21). The same qualified neuropsychologist gave the tasks to all participants, in identical order. The examiner was blind to the participants' diagnoses.

#### Visuo-spatial Memory

The Paired Associate Learning (PAL) task was administered. PAL is a widely used task for visuo-spatial memory and learning, proposed as a marker of hippocampal dysfunction (22). It requires subjects to learn the location of specific visual patterns and may be considered as a proxy of hippocampal functional integrity. Designs are presented in boxes on the screen at varying locations. The designs are then presented sequentially in the center of the screen and subjects are instructed to indicate the box in which each design was initially presented. The task involves sequential stages of increasing difficulty. The total number of errors adjusted for the number of stages successfully completed (“total errors adjusted”) was recorded.

#### Executive Function

The following tasks were administered:

1. The Stockings of Cambridge (SOC), which investigates the ability of planning and problem solving and may be considered as a proxy of prefrontal lobe functional integrity (19). It represents a modified version of the well-known Tower of London task and requires subjects to rearrange colored balls in vertical columns to match a desired final arrangement in a specified minimum number of moves. Subjects are asked to plan their sequence of moves before starting to move the balls shown on the monitor. “Total problems solved in minimum moves” was recorded as the basic measure of the subject's planning ability.
2. The Intradimensional/Extradimensional Attentional Set Shifting (ID/ED), that involves sequential stages of increasing difficulty and was designed to examine component executive function processes (rule discovery and reversal) evaluated in aggregate by the Wisconsin Card Sorting Test (WCST) (23). ID/ED assesses the ability to maintain attention to different examples within a stimulus dimension and the ability to shift attention to a previously irrelevant stimulus dimension. The number of stages completed as well as the number of errors committed (adjusted for the number of stages successfully completed) were recorded.
3. The Stop Signal Test (SST), which is a test of response inhibition that gives a measure of an individual's ability to inhibit a pre-potent response (24). Subjects are required to press the left or right button when they are shown a horizontal arrow pointing either to the left or to the right, respectively, but to suppress (“stop”) their action if they hear an auditory signal (beep) which is presented at varying time intervals just after the visual stimulus. The time interval between visual and auditory signal at which the subjects successfully suppress their action on 50% of the trials (“stop signal reaction time,” SSRT) was recorded.

### Statistical Analysis

We carried out statistical analyses with SPSS version 25.0. We evaluated the normality of the distribution of all variables with the Shapiro-Wilk test. Differences between the three groups on clinicodemographic variables were assessed with the Kruskal-Wallis (KW) test or one-way ANOVA, as appropriate; differences between the two patient groups were subsequently investigated with *t*-test or Mann Whitney (MW) test, as appropriate. As all neuropsychological measures had intensely skewed distributions not amenable to common transformations to approach normality, we rank-normalized them using Blom's formula (25). Then, differences between groups on neuropsychological measures were tested with ANOVA models. Statistical significance was set at the 0.05 level.

Finally, we used a forward stepwise discriminant function analysis, in which the status of the patient (BD or BPD) was the dependent variable and the rank-normalized neuropsychological tests were the independent predictor variables, in order to produce a linear combination of a subset of the predictors that would most efficiently discriminate between the two groups. This analysis was carried out with Statistica.

## Results

### Clinicodemographic Data

The three groups consisted of 29 BD patients, 27 BPD patients and 22 HC. No statistically significant differences were observed between groups on education but a marginally significant difference in age was found between the two patient groups (Table 1). However, age was not significantly correlated with any neuropsychological measure both in the total sample and in any participant subgroup.

**Table 1:** Demographic and clinical profile of patients with Bipolar Disorder (BD), Borderline Personality Disorder (BPD), and Healthy Controls (HC).

	**BD**	**BPD**	**HC**	**p**	**p**
	**(*n* = 29)**	**(*n* = 27)**	**(*n* = 22)**	**(across3 groups)**	**(BD vs. BPD)**
Age	39.9 ± 8.2	35.4 ± 9.6	37.7 ± 9.4	0.12^a^	**0.04** ^b^
Education	11.5 ± 3.5	11.8 ± 3.1	12.1 ± 3.2	0.81^c^	0.74^d^
HAMD-17	12.1 ± 4.3	11.2 ± 5.3	na	-	0.54^b^
YMRS	6.2 ± 5.9	5.6 ± 4.6	na	-	0.81^b^
Hospitalizations	2.5 ± 1.1	3.1 ± 1.2	na	-	0.70^b^
Pharmacotherapy	(*n* = 26)	(*n* = 27)			
Atypical antipsychotics	22 (85%)	18 (67 %)	na	-	0.20^e^
Lithium	3 (12%)	1 (4 %)	na	-	0.35^e^
Anticonvulsants	16 (62%)	17 (63 %)	na	-	1.00^e^
Antidepressants	15 (58%)	17 (63 %)	na	-	0.78^e^
Benzodiazepines	13 (50%)	14 (52 %)	na	-	1.00^e^

*Mean ± SD or N (%) is presented for each variable by group*.

*HAMD-17, Hamilton Depression Scale-17 items; YMRS, Young Mania Rating Scale*.

a *Kruskall-Wallis*.

b *Mann-Whitney*.

c *One-way ANOVA*.

d *t-test*.

e *Fisher's exact*.

*Bold p < 0.05*.

Our sample's clinical characteristics are also shown in Table 1. The two patient groups displayed no significant differences on HAMD-17 and YMRS scores. For BD patients, illness duration was 14.5 years (SD ± 9.6) with an age of onset at 25.4 years (SD ± 7.6). The respective parameters were not measured in BPD patients since a definite starting point cannot be determined with accuracy for a personality disorder.

All patients were receiving pharmacotherapy (atypical antipsychotics, lithium, anticonvulsants, antidepressants, benzodiazepines) at the time of testing but the two patient groups did not differ in any drug category (Table 1).

### Neuropsychological Performance

The differences of rank-normalized outcomes between groups are summarized in Table 2. All differences across the three groups were significant, with the exception of SSRT. BD patients performed significantly worse than HC on all tests; the largest differences were recorded on SOC (*p* < 0.001), followed by PAL (*p* = 0.002), SST (*p* = 0.016), and ID/ED (*p* = 0.020). BPD patients performed significantly worse than HC on all tests except SST (*p* = 0.15); the largest differences were recorded on ID/ED (*p* < 0.001), followed by PAL (*p* = 0.012), and SOC (*p* = 0.013). Significant differences between the two patient groups were recorded only on ID/ED, with BPD patients performing worse (*p* = 0.044); BD patients performed worse on all remaining tests but the difference was not statistically significant (Table 2). Age or medication-adjusted comparisons of rank-normalized neuropsychological measures across groups produced similar results (Supplementary Tables 1, 2).

**Table 2:** Neuropsychological profile of patients with Bipolar Disorder (BD), Borderline Personality Disorder (BPD), and Healthy Controls (HC).

	**BD**	**BPD**	**HC**	***p***	***p***	**Hedge's g (95% CI)**
	**(*n* = 29)**	**(*n* = 27)**	**(*n* = 22)**	**(across3 groups)**	**(BD vs. BPD)**	**(BD vs. BPD)**
PAL errors	46.71 ± 44.5	36.56 ± 33.3	15.77 ± 15.7	0.005^**^	0.51	0.18 (−0.35, 0.71)
ID/ED errors adjusted	23.64 ± 16.2	48.16 ± 38.7	16.14 ± 10.9	0.0003^***^	0.044^*^	−0.55 (−1.08, −0.001)
SOC problems solved	6.11 ± 1.57	6.91 ± 1.67	7.53 ± 1.31	0.001^**^	0.21	−0.34 (−0.87, 0.20)
SSRT	234.5 ± 128.7	216.6 ± 151.4	173.4 ± 33.7	0.053	0.43	0.21 (−0.31, 0.73)

*Mean ± SD is presented for each variable by group*.

*All comparisons were performed in ANOVA models for rank-normalized CANTAB measures*.

*PAL, Paired Associates Learning; ID/ED, Intra dimensional/Extradimensional Set Shifting; SOC, Stockings of Cambridge; SSRT, Stop Signal Response Time*.

* *p < 0.05*,

** *p < 0.01*,

*** *p < 0.001*.

The rank-normalized neuropsychological tests of the two patient groups were then included in a forward stepwise discriminant analysis. The final model was based on 53 patients with complete data on all variables (28 bipolar, 25 borderline). This model was statistically significant (*p* = 0.027) and included two predictors: ID/ED errors adjusted and SOC problems solved (Table 3). The strongest predictor was ID/ED errors adjusted, confirming the results of the previous analysis (Table 2). The classification matrix showed that the model predicted correctly patients' group at 67.9 % of cases (Table 4).

**Table 3:** Forward stepwise discriminant function analysis (BD vs. BPD patients).

**Step**	**Variables**	**F-Remove(1, 51)**	***p***	**Tolerance**	**Wilks' Lambda**
1	ID/ED	4.266	0.044	1.000	0.926
2	ID/ED	6.052	0.017	0.905	0.865
	SOC	3.536	0.066	0.905	

*Final step of forward stepwise model:*

*Wilks' Lambda: 0.865, F_(2, 50)_ = 3.895, p = 0.027, Canonical correlation = 0.367*.

*ID/ED, ID/ED errors adjusted; SOC, SOC total problems solved in minimum moves*.

*Neuropsychological measures were rank-normalized using Blom's formula before inclusion*.

**Table 4:** Classification matrix from final discriminant analysis model.

**Observed group**	**Percent correct**	**Predicted group**	
		**Bipolar patient (prior *p* = 0.53)**	**Borderline (prior *p* = 0.47)**
Bipolar patient (*N* = 28)	67.9	19	9
Borderline (*N* = 25)	68.0	8	17
Total (*N* = 53)	**67.9**	27	26

*Bold value indicate correct prediction of group*.

## Discussion

We examined the differences on neuropsychological parameters among female patients with BD and with BPD using a group of healthy controls for comparison. The parameters tested pertained to paired associates learning, set shifting, problem solving, and inhibition control. BD patients performed significantly worse than controls on all tasks. BPD patients performed significantly worse than HC on all tests except SST. The two patient groups did not differ from each other on paired associates learning, problem solving and inhibition control. BPD patients performed worse on the set shifting executive function test.

We found memory deficits on the associative memory task (PAL) in both BD and BPD patients in comparison with the HC group; this is in accordance with relevant studies (11, 13). This could imply a specific impairment in the ability to learn random visuospatial associations. Given that the PAL test is mainly dependent on the integrity of the hippocampal region, the patients' performance may suggest a dysfunction of this area (22). This could be due to the harmful effect of the hypercortisolaemic state, often associated with (both) BD and BPD (26, 27). High level of circulating glucocorticoids may have damaged the hippocampal area and its connections with other brain areas involved in memory formation and consolidation. The hypothesis that this would affect BD patients more was not confirmed (BD vs. BPD effect size 0.18) but our study was underpowered to detect small or moderate effect size differences.

The findings concerning executive function indicated that both patient groups differed from controls. While direct comparison between BD and BPD showed that they also differed though not significantly in problem solving (effect size 0.34), BPD compared to BD patients showed the largest deficits in attentional set shifting (effect size 0.55), especially at the extra-dimensional shift stage. Several studies have supported that BPD patients compared to HC show more deficits in executive functions; this has been suggested as a marker of impaired fronto-limbic connectivity which could explain the impulsivity seen in patients with BPD (28). Dysregulation of the dorsolateral prefrontal cortex in response to negative emotional stimuli has also been reported in BPD patients compared to controls (29). Mahli et al. (30) have shown that fronto-limbic dysfunction is present in both BDP and BD, underlining some key differences: BD patients require additional top-down cognitive processing to regulate emotion (through the activation of frontal areas) whereas BPD patients have insufficient bottom-up feedback (as shown by the diminished amygdala activity). Furthermore, functional imaging studies (PET) have shown that patients with BPD present glucose hypometabolism in the dorsolateral frontal cortex and the limbic system (anterior cingulate cortex) compared to normal controls, suggesting that the disorder may stem from a failure of the prefrontal cortex to regulate the limbic system (31).

Gvirts et al. (6) directly compared executive function of BD and BPD patients using CANTAB. They found that both BD and BPD patients performed worse than controls in attention and problem solving but they did not find differences between patients and controls in set shifting. However, they used norms of matched healthy controls, while we have included a control group with a similar demographic profile. Concerning between-patients comparison, they found that BD patients had more deficits in a working memory test (as an index of poor utilization of strategy) while we have not found significant deficits for the BD group in paired associates learning (we have used a test that examines “learning memory” more than “executive memory”). Furthermore, they reported differences in times concerning problem solving, while we have not. Similarly to us, they did not find statistical differences in the number of problems solved (6). A main difference from our study is that they did not find differences in set shifting, while we found that BPD patients performed worse in extradimensional shifting.

We expected inhibition control to be one of the tests that BPD patients would have shown more deficits as impulsivity is a core characteristic of BPD. On the contrary, BPD and BD patients had similar performance in inhibition control (BD vs. BPD effect size 0.21), although only BD patients performed significantly worse than HC. The literature findings comparing BD and BPD are controversial at this point given that BD patients display high levels of impulsivity too (2). Our study suggests that deficits in inhibition control cut across BD and BPD diagnoses.

Although neuroimaging studies seem promising, they cannot positively link neuropsychological function to certain brain regions. Based on clinical, neurobiological, neuropsychological and neuroimaging studies, the literature remains inconclusive on the overlap between BD and BPD (2–4). The features that rather connect, than distinguish BD and BPD are childhood trauma, mood instability, and impulsivity. Our effort to find neuropsychological differences between BD and BPD showed that they share a similar profile, with the exception of set shifting.

As regards the limitations of our study, all patients were females: this restricts the results only to women, but could provide a framework for future within-sex comparisons among BD and BPD patients. The sample was relatively small thereby limiting our power to detect differences with small to moderate effect size, but the groups were matched on various characteristics. All patients were hospitalized, but were examined in at least partial remission close to discharge and no significant differences on depression and mania scales were detected across groups. Finally, all patients were under medication, but similar kind of psychotropic drugs were used for the two patient groups. CANTAB is an easily administered neuropsychological tool, although it has been proposed that it may have some shortcomings (high cost, misinterpreting findings, over-diagnosis, and absence of clinician-patient interaction) (32).

In conclusion, bipolar and borderline personality female patients appear to be more similar than different as regards their neuropsychological functions. Our study was the first to show that patients with borderline personality disorder appear more impaired in set shifting, a marker of cognitive flexibility. Our results tentatively suggest a considerable overlap among the two clinical entities at least in females. In other words, the question of whether borderline personality disorder could in fact be positioned inside the bipolar spectrum—in women at least-, remains open to debate.

## Data Availability Statement

The raw data supporting the conclusions of this article will be made available by the authors, without undue reservation.

## Ethics Statement

The studies involving human participants were reviewed and approved by Ethics Comittee of Attikon University Hospital. The patients/participants provided their written informed consent to participate in this study.

## Author Contributions

IM was a co-designer of the study and wrote the manuscript. KT and AK participated in data collection and processing and wrote the manuscript. AP and RG revised the manuscript. NS contributed to the statistical analysis and interpretation of the data. PF was a co-designer of the study, contributed to the statistical analysis, drafted, and made critical and substantial corrections in the manuscript. All authors read and approved the final manuscript.

## Conflict of Interest

The authors declare that the research was conducted in the absence of any commercial or financial relationships that could be construed as a potential conflict of interest.

## Publisher's Note

All claims expressed in this article are solely those of the authors and do not necessarily represent those of their affiliated organizations, or those of the publisher, the editors and the reviewers. Any product that may be evaluated in this article, or claim that may be made by its manufacturer, is not guaranteed or endorsed by the publisher.
