# Supplementary material for: Similar or Different Neuropsychological Profiles? Only Set Shifting Differentiates Women With Bipolar vs. Borderline Personality Disorder
Source: Front Psychiatry. 2021 Jul 28;12:690808. doi: 10.3389/fpsyt.2021.690808 (PMC8355351; doi:10.3389/fpsyt.2021.690808)
Supplement: Supplementary file 1 [file Table_1.DOCX]

**Suppl. Table 1.** Age-adjusted comparisons of patients with Bipolar Disorder (BD), Borderline Personality Disorder (BPD) and Healthy Controls (HC) on their neuropsychological profile.

|  | BD  (n=29) | BPD  (n=27) | HC  (n=22) | p  (across  3 groups) | p  (BD vs. BPD) | Partial η^2^  (BD vs. BPD) |
| --- | --- | --- | --- | --- | --- | --- |
| PAL errors | 46.71±44.5 | 36.56±33.3 | 15.77±15.7 | 0.005** | 0.79 | 0.001  (small) |
| ID/ED errors adjusted | 23.64±16.2 | 48.16±38.7 | 16.14±10.9 | 0.0002*** | 0.04* | 0.082 (medium) |
| SOC problems solved | 6.11±1.57 | 6.91 ±1.67 | 7.53±1.31 | 0.001** | 0.24 | 0.028  (small) |
| SSRT | 234.5±128.7 | 216.6±151.4 | 173.4±33.7 | 0.064 | 0.74 | 0.002  (small) |

Mean ±SD is presented for each variable by group.

All comparisons were performed in ANCOVA models for rank-normalized CANTAB measures adjusting for age.

PAL: Paired Associates Learning, ID/ED: Intra dimensional/Extradimensional Set Shifting, SOC: Stockings of Cambridge, SSRT: Stop Signal Response Time.

* p<0.05, ** p<0.01, ***p<0.001.

**Suppl. Table 2.** Effects of diagnosis and medication on patients’ neuropsychological profile.

|  | PAL errors | ID/ED errors adjusted | SOC problems solved | SSRT |
| --- | --- | --- | --- | --- |
| Diagnosis (BPD=1, BD=0) | b=-0.41, p=0.20 | b=0.68, **p=0.026** | b=0.26, p=0.38 | b=-0.22, p=0.47 |
| Atypical antipsychotics | b=-0.38, p=0.31 | b=0.24, p=0.49 | b=0.06, p=0.85 | b=0.34, p=0.35 |
| Lithium | b=0.44, p=0.41 | b=0.36, p=0.49 | b=0.17, p=0.73 | b=0.30, p=0.58 |
| Anticonvulsants | b=-0.52, p=0.16 | b=0.51, p=0.15 | b=-0.55, p=0.12 | b=-0.10, p=0.78 |
| Antidepressants | b=0.26, p=0.39 | b=0.33, p=0.25 | b=0.51, p=0.08 | b=0.41, p=0.16 |
| Benzodiazepines | b=-0.07, p=0.78 | b=-0.25, p=0.34 | b=0.01, p=0.95 | b=0.04, p=0.90 |

BD, Bipolar Disorder; BPD, Borderline Personality Disorder

Multiple linear regression models were performed for rank-normalized CANTAB measures with diagnosis and medication categories as predictors.

PAL: Paired Associates Learning, ID/ED: Intra dimensional/Extradimensional Set Shifting, SOC: Stockings of Cambridge, SSRT: Stop Signal Response Time.

Bold, p<0.05
